# “Microbiota, symbiosis and individuality summer school” meeting report

**DOI:** 10.1186/s40168-020-00898-7

**Published:** 2020-08-14

**Authors:** Isobel Ronai, Gregor P. Greslehner, Federico Boem, Judith Carlisle, Adrian Stencel, Javier Suárez, Saliha Bayir, Wiebke Bretting, Joana Formosinho, Anna C. Guerrero, William H. Morgan, Cybèle Prigot-Maurice, Salome Rodeck, Marie Vasse, Jacqueline M. Wallis, Oryan Zacks

**Affiliations:** 1 Columbia University, 1200 Amsterdam Ave, New York, 10027 NY USA; 2 ImmunoConcept, UMR5164, CNRS & University of Bordeaux, 146 Rue Léo Saignat, Bordeaux, 33076 France; 3 Dipartimento di Filosofia e Scienze dell’Educazione, Università degli Studi di Torino, Palazzo Nuovo, Via Sant’Ottavio, 20, Torino, 10124 Italy; 4 Washington University in St. Louis, Department of Philosophy, One Brookings Drive, St. Louis, 63130-4899 MO USA; 5 Institute of Philosophy, Jagiellonian University, Grodzka 52, Kraków, 33-332 Poland; 6 Abteilung Philosophie, Universität Bielefeld, Universitätsstraße 25, Bielefeld, 33615 Germany; 7 Institut für Philosophie,Universität Kassel, Henschelstr. 2, Kassel, 34127 Germany; 8 Medical Museion, Department of Public Health, University of Copenhagen, Fredericiagade 18, Copenhagen, 1310 Denmark; 9 Arizona State University, Center for Biology and Society, 427 E Tyler Mall, Tempe, 85281 AZ USA; 10 The University of Sheffield, Department of Philosophy, 45 Victoria Street, Sheffield, S3 7QB UK; 11 Université de Poitiers, Laboratoire Écologie et Biologie des Interactions, UMR CNRS 7267, Bâtiment B35, 5 rue Albert Turpain, TSA 51106, Poitiers Cedex 9, 86073 France; 12 Leibniz Center for Literary and Cultural Research, Schützenstr. 18, Berlin, 10117 Germany; 13 Institute for Integrative Biology, ETH Zürich, Universitätstrasse 16, Zürich, 8092 Switzerland; 14 University of Bristol, Department of Philosophy, Cotham House, Bristol, BS6 6JL UK; 15 Sagol School of Neuroscience, Tel Aviv University, Tel Aviv, 6997801 Israel

**Keywords:** Microbiome, Holobiont, Hologenome, Philosophy of biology, History of biology, Downward causation, Ecology, Conceptual analysis, Holistic, Physiological individuals

## Abstract

How does microbiota research impact our understanding of biological individuality? We summarize the interdisciplinary summer school on “Microbiota, symbiosis and individuality: conceptual and philosophical issues” (July 2019), which was supported by a European Research Council starting grant project “Immunity, DEvelopment, and the Microbiota” (IDEM). The summer school centered around interdisciplinary group work on four facets of microbiota research: holobionts, individuality, causation, and human health. The conceptual discussion of cutting-edge empirical research provided new insights into microbiota and highlights the value of incorporating into meetings experts from other disciplines, such as philosophy and history of science.

Video Abstract

Video Abstract

## Introduction

**Fig. 1:**
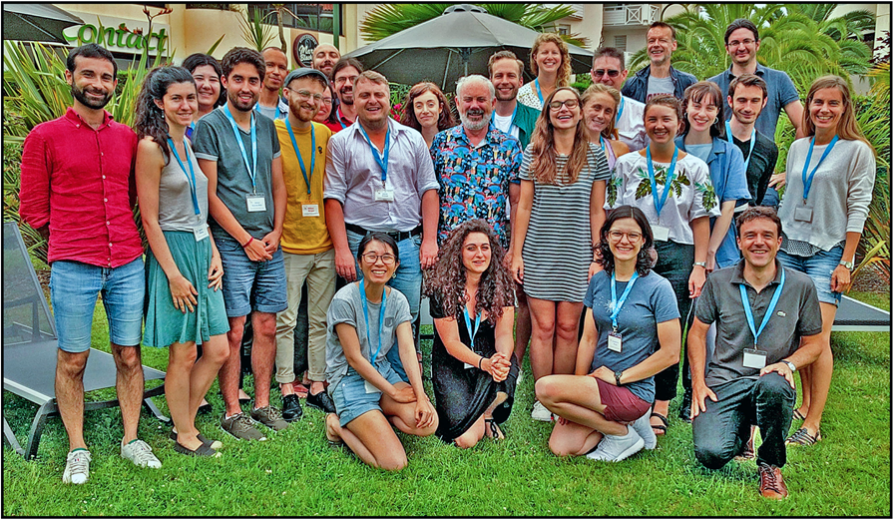
All participants of the summer school. Front row (*from left to right*): Linh-Phuong Nguyen, Cybèle Prigot-Maurice, Jacqueline M. Wallis, Thomas Pradeu. Back (*from left to right*): Guglielmo Militello, Marie Vasse, Isobel Ronai, Javier Suárez, Matt Sims, William H. Morgan, Joana Formosinho, Federico Boem, Gregor P. Greslehner, Adrian Stencel, Saliha Bayir, Scott Gilbert, Malthe Kouassi Bjerregaard, Oryan Zacks, Judith Carlisle, Anna C. Guerrero, Rob Knight, Jan Pieter Konsman, Alice Beck, Salome Rodeck, Quentin Hiernaux, Johannes Martens, Wiebke Bretting

The “Microbiota, symbiosis and individuality: conceptual and philosophical issues” interdisciplinary summer school (1–5 July 2019) in Biarritz (France) explored how microbiota research impacts our conception of biological individuality. The summer school brought together twenty early career researchers and six world-leading experts, across multiple disciplines (biology, philosophy of science, and history of science), with an interest in microbiota and individuality (Fig. 1). This gathering was funded by the European Research Council through a Starting Grant to Thomas Pradeu for the project “Immunity, DEvelopment and the Microbiota—Understanding the Continuous Construction of Biological Identity” (IDEM).

In light of the microbiota “revolution,” there is increasing recognition that the construction of biological identity includes a dynamic dialog with an organism’s microbiota and is dependent on environmental factors [1–5]. A re-examination of biological identity and individuality is therefore needed. How are biological identity and individuality constructed, what kind of phenomena are they, and what are the implications for science and biomedicine [6–8]? A fruitful way to inform biological and biomedical discourse is to engage philosophers and historians of science with the empirical research [9, 10]. Philosophers and historians of science can act as “productive disrupters,” by embedding scientific research in its socio-historical context, offering conceptual analysis of ongoing research, bridging between different knowledge domains, tracing and revealing underlying ontological commitments, and articulating the consequences of alternative epistemologies.

The goals of the summer school were to (i) examine working definitions of the terms used in microbiota research; (ii) clarify the exact sense of the terms “individuality” and “identity,” including how they are impacted by microbiota research; (iii) clarify which domains of the biological sciences, medical sciences, and humanities can be combined to catalyze exploration of connections between microbiota and individuality questions; and (iv) generate future interdisciplinary collaborations on the topic of microbiota.

The potential disciplinary barriers among participants from different academic backgrounds required the summer school to be carefully structured. The meeting consisted of an interdisciplinary reading list, plenary lectures by the six course leaders (summarized in Table 1) and participant-driven interdisciplinary group work. Each group explored a particular facet of microbiota research: holobionts, individuality, causation, and human health. Our meeting report focuses on the open questions that arose for each of these four topics, as well as the tentative answers offered in response to these questions. We showcase interdisciplinary movements of thinking, which we believe will be helpful for advancing microbiota research.

**Table 1:** Plenary lectures from course leaders

**Presenter,**	**Lecture title**	**Description**	**Associated**
**affiliation**			**references**
Thomas Pradeu,	Microbiota and	Pradeu asked “what do we mean by microbiota?” and	[2, 17]
The French National	microbiome: a	pointed out there is no single answer. He also argued that	
Center for Scientific	conceptual analysis	our understanding of microbiota impacts our conception of	
Research and University		individuality. Examining the history, meaning, and impact of	
of Bordeaux (France)		the microbiota is important when making ontological and	
		epistemological claims concerning individuality.	
	Interactions between	Pradeu highlighted how the function of the immune system	[5, 18–20]
	the microbiota and the	has been reconsidered in light of microbiota research. The	
	immune system: an	immune response should be thought of as a dynamic	
	immunological point of	equilibrium, regulated by activating and inhibitory signals as	
	view on biological	a function of the ecological context and the encountered	
	individuality	microbes. Pradeu proposed a physiological individual as a	
		unit of functioning, composed of the host and its microbiota,	
		where the immune system plays a crucial role in the	
		unification of this plurality.	
Scott Gilbert,	Developmental symbiosis	Gilbert argued that all metazoans have microbial symbionts	[4, 21, 22]
Swarthmore College	and the mapping of	and these are important, sometimes essential, for normal	
(USA) and University	novel evolutionary	animal development and organ generation. For example, the	
of Helsinki (Finland)	trajectories	gut of cows has been transformed by symbionts and led to	
		the emergence of their herbivory diet. The close association	
		of organisms and their microbiota therefore opens novel	
		evolutionary trajectories. Organisms have been formed by	
		symbiotic interactions and these close associations open	
		novel evolutionary trajectories.	
Johannes Martens,	Biological individuality:	Martens provided philosophical context for the concept of	[23, 24]
University Catholique	a conceptual analysis	biological individuality. He distinguished it from other	
of Louvain (Belgium)		concepts, such as unity, and argued that questions of	
		individuality primarily involve singling out the properties that	
		make an individual distinct. Productive theorizing about	
		individuality does, of course, require considering individuals	
		themselves, but it also involves considering their parts, as well as	
		the collectives they form.	
	Fraternal vs.	Martens argued that there are two concepts associated with	
	egalitarian transitions in	transitions in biological individuality. First, fraternal transitions	
	individuality: two	involve a transition in Darwinian individuality (e.g., multicellularity	
	processes, one concept?	and insect colonies). Second, egalitarian transitions involve a transition	
		in organismality, where the entities share a dependence and mutual	
		benefit (e.g., the eukaryotic cell). The identification of two concepts	
		for major transitions is helpful for exploring the influence of holobionts	
		on evolution at multiple levels of biological organization.	
Jan Pieter Konsman,	Barriers and	Konsman argued that we ought not confuse the existence of	[25, 26]
The French National	obstacles in relation	a functional “axis” between the microbiota, host gut, and	
Center for Scientific	to microbiota’s host	host brain with the presence of precise mechanistic	
Research and	effects	interactions between the organisms involved in this axis	
University of Bordeaux		(which remain largely unconfirmed). The biological barriers	
(France)		have a dynamic nature and act more like borders, localized	
		areas over which complex regulation and interaction occurs.	
		Konsman concluded that methodologies and explanations	
		must consider host organization and other higher-level	
		features which can both inform and structure the	
		reductionistic methodologies present in biology.	
Thomas Bosch,	The holobiont	Bosch argued that biology and medicine have historically	[11, 27, 28]
University of Kiel	imperative: towards	focused on the host, missing the important role of the	
(Germany)	a holistic understanding	microbiota. Using his experimental work on the Hydra	
	of complex life processes	metaorganism as a model system for the evolution of	
		biological complexity, Bosch concluded that the	
		metaorganism perspective invites a more holistic and	
		integrative account of an organism.	
Rob Knight,	Beyond the tip of	Using his research investigating the impact of microbiota on	[29–33]
University of	the iceberg:	human health, Knight argued that microbiota research needs	
California, San	discovering millions	to focus more on determining causal pathways, examining	
Diego (USA)	more “human”	the transgenerational effects of microbiota and intervening	
	genes in our	on the microbiota. On the other hand, even without these	
	microbiomes and	possible advances, current microbiota research is already	
	their links to	challenging classical philosophy of biology debates—	
	phenotype	including debates about phenotypes and evolution, as well as	
		what counts as a unit of selection.	

## Microbiota and the holobiont: can we understand the holobiont in isolation from its ecological boundaries?

“Holobiont” is a biological concept that has received considerable attention. However, its definition is highly contested and somewhat convoluted, casting doubt on its theoretical or practical usefulness. The concept can be defined as “an association comprised of the macroscopic host and synergistic interdependence with bacteria, archaea, fungi, and numerous other microbial and eukaryotic species” (Table 1 Bosch’s lecture; [11]). The holobiont concept aims at emphasizing the importance of symbiotic relationships for an organism. Being more than the sum of its parts, as one participant group argued, the holobiont is a totality of complex relationships between different biological entities [8].

A major problem with the concept of the holobiont is how to determine its ecological boundaries: should the holobiont encompass the host plus the totality of its microbes, or are the microbes part of the environment of the host? To answer this question, one participant group examined different case studies from research into symbioses. For example, the symbiosis of the Hawaiian bobtail squid (*Euprymna scolopes*) and bacteria *Vibrio fischeri* enable the holobiont to have a light organ [12]. Another example comes from coral holobionts [13, 14]. Soft corals, such as *Leptogorgia alba*, rely on bacterial symbionts as a defense against pathogenic fungi [15]. When *L. alba* feeds at night, it is susceptible to pathogenic fungi and the bacterial symbiont *Pseudoalteromonas* sp. produce anti-mycotic molecules that protect the holobiont, but only under low-light conditions [16]. These examples suggest that the holobiont’s microbiota can be seen as adapted to the environment along with the host, and the holobiont concept opens up new ways of thinking about the nature of organisms and their boundaries.

There is a complex relationship between the microbial cells that compose the microbiome, and their host cells, from which they diverge genetically [1, 11]. The emerging consensus is that symbiotic microbes function in a similar way to host cells rather than as an aspect of the external environment, because they perform functions that were previously ascribed only to host cells. For example, microbiota allowed the evolution of herbivory through specialized digestion (Table 1 Gilbert’s lecture; see also [4, 22]) and microbiota facilitate functionality of the immune system (Table 1 Pradeu’s lecture; see also [17]). Importantly, this happens regardless of the genetic difference between host cells and microbial cells. Both examples, therefore, underscore the importance of the holobiont concept as a guiding research tool in contemporary biology.

Thus, using the holobiont concept as only a shorthand for a “multicellular host plus its microbes” limits its potential, if the interactions between these elements are not taken into account too. The most important features of the concept are its power to render tangible the fundamental interdependence of all living beings and complexity of organismic life. The history of science teaches us that some biological concepts might be distorted or misunderstood but still have a positive impact on research by generating progressive research methods [34, 35]. The emerging field of holobiont research highlights the benefits of a holistic understanding of life and its research methods study the holobiont in its entirety.

## Microbiota and individuality: does microbiota research affect our understanding and definition of a physiological individual?

What counts as an individual is question-dependent as different research contexts have different ways of characterizing individuality (Table 1 Pradeu’s lecture; Gilbert’s lecture; [4, 17, 36]). Some of the suggested conceptions of biological individuality have been evolutionary, ecological, immunological, and developmental [9, 17, 37, 38]. Holobiont research presents a unique challenge to the traditional evolutionary conceptions of biological individuals. These traditional conceptions used a set of criteria based on biological terms such as heritability and selection [2], which seems to exclude holobionts. For example, Godfrey-Smith’s oft-cited evolutionary account defines Darwinian individuals in terms of variation in heritable traits resulting in different reproductive advantages across generations [39]. There is a debate whether symbiotic relationships between organisms and their microbiota satisfy the evolutionary criteria for individuality because they often fail to collectively show variation, heritability, and differences in reproductive success [40–43].

Holobionts appear intuitively “individualistic” because its constituent organisms often cannot survive without one another, and they are structurally, metabolically, developmentally, and immunologically integrated. Thus, holobionts may constitute a new conception of biological individuality. The need for a functionally relevant term to capture the holobiont as a well-delineated and cohesive unit led a participant group to propose that holobionts are physiological individuals (Table 1 Martens’ lecture). A physiological individual is characterized by the functional integration of metabolism and immune activities.

It is difficult to successfully characterize what entities are “physiological individuals.” Some definitions seem to either exclude entities that should be physiological individuals (for example, plants) or include entities that are not physiological individuals (for example, biochemical processes in a lab setting) [9, 17]. The participant group defined the most basic form of physiological individuality in order to relate other biological entities to this basic form in a scalar fashion. A minimal model has only the essential ingredients of a living organism, while it maintains separateness and coherence within its environment. An example of the most basic form of physiological individuality is Gánti’s chemoton [44], his criteria include:

1. A semi-permeable barrier in the form of a membrane, which acts as a minimal form of an interface with the environment and defense (filtering over entry);
2. A self-sustaining metabolic cycle; and
3. Heredity of information with the potential for variation in the form of genes.

The chemoton is meant to describe a hypothetical minimal form of life, and because the description of physiological individuality is scalar, the minimal model can be used in a variety of biological contexts and applied to a wide variety of organisms.

**Fig. 2:**
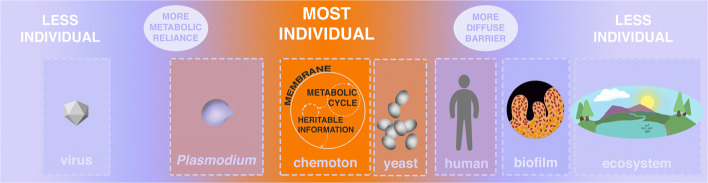
The physiological individuality continuum. The center of the spectrum represents the theoretical paradigm individual, the “chemoton.” Biological entities at the center are maximally individualistic in that they are less metabolically (or genetically) reliant on other organisms, and their barriers are minimally diffuse. As you move out from the center, biological entities become less individualistic, but for different reasons: biological entities on the left side of the spectrum lack individuality due to their metabolic (and/or genetic) reliance on other organisms. Biological entities on the right side of the spectrum lack individuality due to their diffuse barriers

The chemoton can be placed at the center of a “physiological individuality spectrum,” as an ideal but theoretical model of coherence and functional unity. The organisms that most closely show this coherence are single-celled organisms, although they are still highly interconnected with other entities in their environments. The more complex organisms become, the more they tend to “outsource” or engage in relationships of interdependence with other organisms (outside of their own membrane). If Gánti’s model is taken as a paradigm, holobionts no longer meet the minimal criteria for physiological individuality because their barriers become more diffuse, and they interact with other species for metabolism and heredity. In addition, biofilms and symbionts are not counted as physiological individuals due to increasingly “open” barriers. At the other end of the continuum are entities such as viruses, which are highly dependent on other organisms for both metabolic and hereditary processes. The multi-cellular world can therefore be understood as a continuum of interacting organisms displaying different degrees of separateness and interdependence (Fig. 2). A minimal model approach avoids worries about both anthropocentrism and disciplinary isolationism [17, 45].

It is important to note that individuality can be conceived at multiple scales of the biological hierarchy. For example, in a holobiont the relationship between a host and its microbes is intimate, but in an organism the relationship of a cell and its mitochondria can be considered more intimate still. The placement of an entity on the hierarchy of life can help predict consequences of an unraveling of relationships, such as the degree of interdependence we expect to find between its component parts.

## Microbiota and causation: should microbiota research consider downward causation?

Contemporary research suggests that the microbiota have a substantial influence on their multicellular hosts, including host physiology and host immunology (Table 1 Pradeu’s lecture; Bosch’s lecture; [46]). These findings have led biologists to attribute to the microbiota an important causal role in host health, host development, and host evolution (Table 1 Knight’s lecture; Gilbert’s lecture; [47]). However, some biologists and philosophers of biology have persuasively argued that while certain findings show interesting correlations between the microbiota and certain host states, it is not clear that a causal relationship from the microbiota to the host exists [48–51]. Do causal claims in microbiota research require a healthy dose of skepticism?

The methods of microbiota research are usually coarse-grained. These methods are therefore not comparable to the traditional and standardized methods employed to establish causation in other research areas, such as biomedical research. Traditional methods to establish causation are grounded in designing interventions that show a direct connection between an entity and a phenomenon. For instance, one can experimentally show how a pathogen causes a disease using Koch’s postulates [52, 53] or how microbiota affects the physiological functions of their host. While some microbiota therapies cure disease through the inoculation of “healthy” microbiota into “unhealthy” patients (for example, fecal transplantation; see “Microbiota and health” section), the level of analysis for microbiota research is not precise enough to establish a causal pathway as the agents (microbial taxa) that bring about the cure are never identified. Thus, the gold standard of establishing causation is not often met by microbiota research methods.

Given this issue of causation in microbiota research, one participant group discussed whether the tools of metaphysics might be useful. Metaphysics is the branch of philosophy dedicated to the study of the first principles of reality, including the study of the concept of “causation” and the different forms of causation that may exist in the world [54]. A metaphysical study of “causation” in microbiota research helped identify the type of causal relationships that exist.

One can distinguish two types of causation: downward (top-down) and upward (bottom-up). Biomedical research usually appeals to upward causation, referring to situations where a certain entity (for example, a molecule, a bacterium, a virus) is deemed responsible for provoking a phenomenon or activity at the systemic level of the organism (for example, a disease, a physiological process). Downward causation, on the other hand, refers to situations in which the activities at the systemic level of the organism are responsible for changes in the entities at lower levels of organization [55]. Some metaphysicians have claimed that downward causation occurs in scenarios where the system level generates physico-chemical constraints that significantly decrease the degree of freedom of their component parts [56–59]. A representative case of downward causation in biology is meiotic drive. In a normal process of cell division, it is expected that each allele will be transmitted in a 1:1 proportion. Meiotic drive, however, creates a constraint on cellular division by reducing the degree of freedom of certain alleles, so that the final distribution favors some alleles over others, and the proportion differs from 1:1. Therefore, certain situations in nature can be defined as cases of downward causation.

Microbiota research can be thought of in terms of downward causation. The system (holobiont) generates some constraints that reduce the degree of freedom of its components (microbiota). In this sense, a “healthy” holobiont (see “Microbiota and health” Section) would be one that generates constraints that reduce the exponential growth of the potential pathogens contained in it and, consequently, avoids their pathogenicity. Conversely, an “unhealthy” holobiont is one that fails to constrain pathogens. A more nuanced understanding of causation in microbiota research also shows that studying how the growth of a microbial taxon is constrained by its interactions within a holobiont is more helpful than studying the specific effect of a microbial taxon on a healthy holobiont (i.e., using Koch’s postulates). Therefore, the tools of metaphysics provide an understanding of causation in microbiota research and are even helpful for designing new forms of intervention (see the “Future directions” section). A “healthy” holobiont and the development of microbiota-based therapeutics is feasible if the combinations of microbial taxa that constrain the growth of the pathogen are identified.

## Microbiota and health: is human health a systemic property of the holobiont and does it matter for medical practice?

Human health is intimately intertwined with the ecology of a human’s microbiota. One participant group proposed human health should be conceptualized as a property of the holobiont not just the human. A holobiont is a functional whole whose features are constituted by the relations that occur between its component parts (see “Microbiota and the holobiont” Section). Therefore, the human health needs to address both the systemic-ecological interactions (also known as “emergence”, see “Microbiota and causation” Section) and individual component parts.

If the concept of the holobiont is transferred to a medical context, the current World Health Organization definition of health, as “a state of physical, mental, and social well-being and not merely the absence of disease or infirmity” [60] would therefore be better conceived of as a plural and systemic concept. Health factors are social, biological, cultural, and environmental factors, along with their dynamic interactions. These factors do not belong to a human individual, rather they arise from interactions. These interactions are systemic and ecological, since perturbing them will provoke systemic modifications, adjustments, or disruptions. The altered dynamics of the holobiont system are what, macroscopically, we call “health” and determines the pathological condition. Thus, a holobiontic perspective views human health as arising from complex, locally interactive human, and non-human systems, with multiple balance points occurring over time. Under this perspective, health and illness are not binaries but instead result from potentially overlapping properties of a locally dynamic system. The concept of the holobiont also leads us to modify our understanding of individuality (see “Microbiota and individuality” section). Clinical practice should not neglect the fact that “a single individual” is actually a functional whole of different biomes.

If the holobiont is considered the therapeutic unit this would mean it is the privileged target of therapeutic actions. The manipulation of microbiota will require a serious reflection on manipulation criteria in experimental practice (see “Microbiota and causation” section) and perhaps should be more grounded in ecological knowledge principles [61, 62]. Ecological manipulation of the microbiota is likely to be totally different from traditional, mechanistic interventions and thus requires new theoretical and experimental accounts in order to be successfully employed.

The best case study of a therapeutic approach transitioning to a holobiont perspective is gastrointestinal disease, an infection with the bacteria *Clostridium difficile*. Traditionally, *C. difficile* infections were treated with antibiotics, whose non-discriminatory nature meant that the entire gut microbiota was broadly weakened, and this treatment had a low success rate in curing the disease [63, 64]. A more successful intervention is fecal microbiota transplantation, where fecal matter is taken from a healthy donor and transplanted into the patient [65–67]. This treatment is successful in curing *C. difficile* infections because it is a holobiont-based therapeutic intervention on the systemic-ecological interactions. A diverse gut microbiota can prohibit the invasion of particular (potentially pathogenic) microbial species under colonization resistance theory [68–70]. However, the causal pathways underlying the success of fecal microbiota transplantation are not yet well understood (see “Microbiota and causation” section). Additionally, large inter- and intra-patient variability means that a “healthy microbiota” for one individual is unlikely to be healthy for another [71–73]. A personalized medicine approach to the human microbiota is perhaps needed.

A holobiontic perspective has potential implications on the healthcare structures and practices that impact the systemic-ecological balance of patients. Hygiene practices in modern Western medicine have been based on the idea of an autonomous, delocalized human individual, which appears no longer adequate in light of the holobiont. A holobiontic perspective recognizes that a “sterile environment” is unsafe and ripe for colonization by microbial newcomers. So all microbes should not be removed, rather a protective balance of healthy microbiota ecology should be preserved [61]. The barriers to implementing a holobiont perspective are not just scientific and technological but also societal and cultural. For example, the public perception of microbes needs to be changed and conventional public expectations about sterile environments overturned. The frequently used war-like, host-centered language in medicine, such as “microbes as enemies,” “war on X,” and “fighting disease” (Table 1 Pradeu’s lectures), should either be highly revised or abandoned.

## Future directions

Microbiota research is changing our understanding of the ecological boundaries of holobionts and what it means to be an individual in terms of causation, physiology, and health. The cross-talk between biology and the philosophy/history of science will continue. We speculate about some of the future impacts on microbiota research here.

Microbiota research raises important questions concerning which species count as part of the holobiont (see “Microbiota and the holobiont” section). Should we consider the host and its microbiota to be a kind of whole, as some suggest [1, 11, 74], and commit ourselves to holistic thinking about holobionts? In this way, we would have to accept that holobionts constitute a genuine kind of biological unit and that they are non-reducible to the mere sum of their parts, insofar as they include the synergies between their components. Talking about the holobiont redirects biology’s focus towards an understanding of nature as being fundamentally symbiotic.

We proposed a physiological individuality spectrum for biological entities, which relies primarily on Gánti’s chemoton as an ideal model of coherence and functional unity (see “Microbiota and individuality” section). This spectrum allows us to highlight the ways that the holobiont is individualistic (e.g., structurally, metabolically, developmentally, and immunologically integrated) while recognizing that some holobionts may not be what has traditionally been called evolutionary individuals [75, 76]. We hope that placing holobionts on this spectrum will provide novel and testable hypotheses. For example, it could be that the degree of interdependence we find between a host and its component parts may be an indicator of the importance of this relationship to the survival of the holobiont as a whole. If so, we may be able to use this spectrum to predict and/or intervene on the consequences of unraveling relationships within a physiological individual or community. We believe that our notion of “physiological individuality” is best understood as one among many helpful theoretical conceptions of individuality. There are evolutionary individuals, physiological individuals, developmentally unified individuals, immunological individuals, and perhaps others. By identifying individuality as a pluralistic concept, we can describe the many varieties of individuality, we see in the biological hierarchy.

Our proposal of downward causation being important for microbiota research (see “Microbiota and causation” section) hopefully inspires new research questions. For example, does a healthy vaginal microbial community influence introduced microbes? The vagina is an acidic environment [77] due to bacteria such as *Lactobacillus sp.* [78]. We hypothesize that if a random bacteria is introduced into a healthy vagina, they will either alter their gene expression to produce an acidifying compound or horizontally acquire a genetic component for the production of acids from the resident bacterial species. These types of experiments would provide substantial evidence for the existence of downward causation from the vaginal microbiota to some of the species of microorganisms that compose it.

A holobiont perspective entails re-conceptualizing the “therapeutic individual” as a more-than-human integrated unit, whose clinical identity is continuously constructed in dialog with its microbiota and environment, in contingent, localized dynamics (see “Microbiota and health” section). A better understanding of these dynamics is required and goes beyond the current mechanistic accounts used in biomedicine. Because ecologists study how perturbations reverberate unpredictably through dynamic ecosystems leading to unexpected outcomes, we propose that the hospitals of the future could include ecologists to use their expertise in designing system-level therapeutic interventions, as it has been argued that holobionts have some properties of ecosystems (e.g., [41, 79]). Therefore, as the holobiont is an object of inquiry that challenges current categories of scientific investigations and methodologies, we need new research areas aimed at investigating holobionts.

## Conclusions

The summer school provided a productive platform for collaboration between researchers from different disciplinary backgrounds, all of whom shared an interest in the complex problems of microbiota. An interdisciplinary endeavor faces many challenges. For example, researchers from different disciplines do not have the same knowledge about a subject, which can make it difficult to find a common language and starting point. In addition, researchers have particular methodologies and ways of investigation, and working with someone from another discipline can be tricky. As a result, people tend to interact more with participants from the same disciplinary background. To promote interdisciplinary collaboration, the organizers of the summer school carefully selected participants: biologists with an interest in philosophy/history of science as well as philosophers/historians of science with an interest in biology.

The integration of science, philosophy of science and history of science is beneficial. Philosophy of biology can help biology [10, 80], and biological case studies are a great source of inspiration for philosophical and historical work. The products of the interdisciplinary participant group work were generally wider in scope and more appealing to a broad audience than the outcomes generated by a single discipline.

The novelty, complexity, and potency of microbiota research requires a global, interdisciplinary perspective when moving forward. To keep this flow of mutual inspiration, we need contexts and practices that link the scattered communities of the natural sciences and humanities. This summer school showed us one successful way to do so, and we hope that this “experiment” will be replicated in the future. There is great scope for productive cooperation, but it takes people equipped with the right tools and enthusiasm to open the door and invite researchers from disparate disciplines into the same room.

## Data Availability

Not applicable.
